# Targeted policy intervention for reducing red meat consumption: conflicts and trade-offs

**DOI:** 10.1186/s40795-022-00570-3

**Published:** 2022-08-16

**Authors:** William H.M. James, Nik Lomax, Mark Birkin, Lisa M. Collins

**Affiliations:** 1 School of Geography and Leeds Institute for Data Analytics, University of Leeds, Woodhouse Lane, Leeds, LS2 9JT West Yorkshire UK; 2 Faculty of Biological Sciences, University of Leeds, Woodhouse Lane, Leeds, LS2 9JT West Yorkshire UK

**Keywords:** Diet, Health, Meat Industry, Climate Change

## Abstract

**Background:**

There are a range of policies and guidelines focused on meat consumption which aim to tackle health and environmental issues. Policies are often siloed in nature and propose universal limits on consumption. Despite this, there will be a number of conflicts and trade-offs between interest groups. This study explores secondary impacts associated with guidelines issued by the World Cancer Research Fund and assesses the utility of a targeted policy intervention strategy for reducing red meat consumption.

**Methods:**

We used highly detailed consumption data of over 5,000 individuals from the National Diet and Nutrition Survey. We firstly compared individual consumption against the policy guidelines to identify demographic groups most likely to consume above recommended levels. We then synthetically modified the food diary data to investigate the secondary impacts of adherence to the recommendations by all individuals. We assessed changes in overall consumption, nutrient intake (iron, zinc, vitamin B12, vitamin B3, fat and saturated fat) and global warming potential. We also projected future impacts under various population projections.

**Results:**

We found that certain demographic groups are much more likely to exceed the recommendations and would therefore benefit from a targeted intervention approach. Our results provide a baseline for which the impacts of any meat substitute diets can be assessed against. Whilst secondary health benefits may be realised by reducing intake of certain nutrients (e.g. fats), negative impacts may occur due to the reduced intake of other nutrients (e.g. iron, zinc). Reduced overall consumption is likely to have implications for the wider meat industry whilst complementary impacts would occur in terms of reduced greenhouse gas emissions. Impacts will be counteracted or maybe even reversed by any substitute products, highlighting the need to carefully consider the suitability and impacts of meat-replacements.

**Conclusion:**

The future structure of the meat industry will depend on how conflicts and trade-offs are addressed and how more holistic policy ideas are implemented. This research provides a framework for using demographic and consumption data to reduce negative trade-offs and improve policy effectiveness.

## Background

Policy recommendations for reducing meat consumption have recently received substantial attention in the scientific literature and media. Universal limits on consumption are often proposed as a method for tackling issues such as cancer risk [1] and climate change [2]. These policies will inevitably result in a range of conflicts and trade-offs concerning issues such as nutrient intake [3] and the wider impacts on the meat industry due to reduced demand [4]. Despite this, policy intervention and their trade-offs are frequently dealt with in silos, with little emphasis on reducing detrimental secondary impacts. The future structure of the meat industry will depend on how trade-offs between issues are addressed and how more holistic policy ideas are implemented. Whilst the nexus of factors associated with the meat industry is often acknowledged, they are rarely addressed simultaneously in a systematic manner. By better understanding this nexus it may be possible to develop targeted policy interventions which deliver the desired outcome while at the same time reducing detrimental trade-offs.

There are a range of policy interventions which may be used to alter dietary patterns, with an excellent review provided by Brambila-Macias et al. [5]. Whilst these policies are often applied universally to an entire population, many can also be focused on specific groups of individuals or geographical locations in the form of a targeted policy intervention (TPI). This approach is used in a variety of situations such as public health where support and engagement is focused on high risk individuals [6]. For example, the UK government has recently announced restrictions on the advertising of foods high in fat, salt and sugar before 9pm, aiming to protect children from developing long-term unhealthy eating habits [7]. TPIs can also be designed to minimise secondary negative impacts among specific groups. For instance, policy developed for minimum unit alcohol pricing in Scotland aims to improve health outcomes for harmful drinkers whilst minimising financial penalties for low consumers [8]. TPIs can also target individuals who have a disproportionate negative impact, such as in the UK car industry where owners of high polluting vehicles are taxed at a much higher rate than their low-emission counterparts [9].

There are a wide range of policies and guidelines related to red meat consumption focusing on various outcomes. For example, the EAT-Lancet Commission recommends limiting red meat consumption to below 98 grams per week for overall environmental sustainability and health reasons [2]. Other guidelines are focused on specific issues, with guidelines by the World Cancer Research Forum (WCRF) directed at reducing bowel cancer prevalence. In this case, the WCRF recommends that individuals should eat very little, if any processed meat and no more than 500g (cooked weight) of red meat per week [10, 11]. These guidelines are similar to those issues by the National Health Service (NHS) in the UK.

This paper provides an assessment of the impact that TPIs which enact recommendations on red and processed red meat could have when applied to the UK population. This study uses the WCRF guidelines to demonstrate the approach but our framework could equally be applied to other recommendations. We demonstrate the implications and trade offs which emerge for nutrient intake, greenhouse gas (GHG) emissions and the wider industry structure in relation to total consumption of red and processed red meat. We model the impact of meat removal from diets, with the resultant datasets forming the baseline upon which any substitutes will be added to.

### World cancer research fund guidelines

The WCRF offers clearly defined and quantitative recommendations for reducing meat consumption in relation to colorectal (bowel) cancer, the third most common cancer worldwide [12]. Red meat presents a bowel cancer risk due to the presence of haem iron and the formation of additional compounds during cooking. Whilst exceptions do exist, processed red meat presents an increased risk due to the presence of additional carcinogenic compounds, the high fat content and often high cooking temperatures compared to non-processed products [13]. A comprehensive meta-analysis by the World Cancer Research Forum (WCRF) concluded that “consumption of processed red meat is a convincing cause of colorectal cancer” and “consumption of red meat is probably a cause of colorectal cance”. The International Agency for Research on Cancer (IARC), an agency of the World Health Organisation (WHO) also considers processed meat as carcinogenic, classifying it as a Group 1 carcinogen [14]. In light of this evidence, the WCRF recommends that individuals should eat very little, if any processed meat and no more than 500g (cooked weight) of red meat per week [10, 11].

If followed, the WCRF recommendations would likely have a wide range of secondary impacts, which may be positive or negative in nature. Limiting meat consumption will reduce nutrient intake, which may result in deficiencies. This is a concern as red meat is often an important source of nutrients such as iron and zinc, for which some demographic groups have pre-existing low intakes of [3, 15, 16]. Conversely, reducing intakes of other nutrients found in read meat (e.g. fats, sodium) may be beneficial for health conditions including cardiovascular disease [17] and type 2 diabetes [18]. Alongside changes in nutrient intake, reduced meat consumption will also reduce product demand, which has the potential to impact the economic and workforce structure. The meat processing industry currently directly employs over 75,000 people and supports more than 50,000 farmers in the UK [4], highlighting the need to consider such impacts. Additionally, reduced meat consumption is likely to be beneficial for environmental concerns such as land use change [19], water pollution [20] and water use [21]. One of the most prominent environmental issues related to the meat industry is greenhouse gas emissions [22, 23], with production of animal-based food (including livestock feed) contributing 57% of total food sector emissions [24].

Overall impacts will also depend on any substitute products used to replace red meat consumption. For example, an increase in zinc deficiency may be counterbalanced or even reversed if red meat is replaced by high zinc concentration products such as oysters [25]. Similarly, emission reductions achieved by limiting red meat consumption will be counterbalanced by those generated by substitute products. However, the net impact is still likely to be reduced emissions as meat production ordinarily produces more emissions per unit of energy compared with plant-based foods due to energy loss at each trophic level [26]. Furthermore, ruminant livestock (i.e. cows and sheep) produce methane, a powerful greenhouse gas [27]. By modelling the dietary impacts of meat removal alone (i.e. without substitution products), we are able to generate a baseline for which any substitutes will be added to. This baseline may be used to identify important criteria required in substitute products (e.g. abundance of a particular nutrient) or to ensure any substitutions do not reverse any positive impacts of meat removal (e.g. if the substitute products have higher emissions than the meat removed).

## Methods

This study uses comprehensive food diary data from the National Diet and Nutrition Survey (NDNS) downloaded from the UK data service [28]. We use these data to investigate the impacts of aligning red and processed red meat consumption with WCRF recommendations. We compared each individual’s original diet (hereafter termed ‘pre-intervention’) to a modified version which fulfills the WCRF requirements (hereafter termed ‘post-intervention’). We investigated impacts on nutrient intake, GHG emissions and total consumption. In order to quantify bulk impacts (especially in relation to GHG emissions and total consumption) we combined our per-capita consumption estimates with national population estimates. We projected our estimates of GHG emissions and total consumption to the year 2050 by using national population projections and assuming meat consumption patterns by age and sex groups remain consistent over time for both the original and modified diets (e.g. a 63 year old male in 2021 would have the same consumption pattern as a 63 year old male in 2050).

### Food diary

The NDNS provides high quality, nationally representative data on the types and quantities of foods consumed by individuals in the UK. It is used by the UK government to monitor progress toward diet and nutrition objectives of UK Health Departments and to develop policy interventions. Unlike alternative food surveys which record food items purchased (e.g. Living Cost and Food Survey), the NDNS records quantities consumed (i.e. the cooked weight and disregarding any wastage). Data from the NDNS is therefore compatible with dietary recommendations which specify limits on quantities to be consumed, such as those issued by the WCRF.

The NDNS is a continuous cross-sectional survey of the general population aged 1.5 years and over living in private households in the UK. A representative sample of approximately 1,000 people (500 adults and 500 children) take part in the survey each year. The NDNS comprises an interview, a 4-day diet diary, physical measurements and a blood and urine sample. In its current form, the NDNS has been carried out since 2008/09 (referred to as ‘year 1’ by the NDNS), with the latest available results available for 2016/17 (year 9). Due to the sample sizes, our analysis was based on pooled data over 5 years (years 5 – 9, 2012/13 - 2016/17). Utilising the most recent 5 years achieves the best trade-off between ensuring a large enough sample for robust analysis whilst minimising the effect of temporal trends in diet. Public Health England and the Food Standards Agency routinely group multiple years of NDNS data [29] whilst secondary analysis has previously been carried out on six years’ worth of grouped data [30]. To account for different sample sizes in each year, we re-weighted the combined data following the methodology published by Public Health England [29].

### Non-meat content in foods

Assumptions regarding non-meat ingredients are recognised as a potential limitation and source of uncertainty when analysing meat consumption statistics [31]. Although the NDNS provides diary data at the individual food item level, some items may contain a variety of meat and non-meat ingredients. For example, a portion of pre-prepared lasagne may contain minced beef alongside vegetables, cereals and other ingredients. To account for this, the original survey disaggregates composite food items into subcategories using the methodology described in Fitt et al. [32]. The original classification scheme contains meat subcategories of beef, pork, lamb and other red meat alongside ‘meat product’ subcategories of sausages, burgers and grilled steaks, offal and processed red meat. Although a composite dish may contain multiple subcategories (e.g. a ‘meat feast’ pizza may include entries for both beef and pork), individual ingredients are mutually exclusive (e.g. a pork sausage will be recorded in the sausage subcategory but not in the pork subcategory). This format was originally developed due to the interest in purchasing behaviours of individual product types (e.g. sausages) as opposed to the type of meat they contain. Whilst this classification scheme is ideal in our study for identifying food products when aligning diets to the WCRF guidelines (where the guidelines refer to the entire product weight and the differentiation is between processed and non-processed red meat products), it is not suited for our analysis of total consumption, nor for assessment of greenhouse gas emissions, where a full differentiation between meat types is needed. In these cases, we need to know whether a meat product (e.g. a sausage) is comprised of pork, beef or lamb etc. To achieve this, we further disaggregate the product subcategories of sausages, burgers and grilled steaks and processed red meat. For any food item originally containing entries for these categories we use the product description provided by the ‘FoodName’ field in the NDNS to assign the appropriate red meat group (beef, lamb, pork, other red meat). Because the original product level categories from the NDNS (sausages, burgers and grilled steaks, offal or processed red meat) may contain non-meat components (e.g. a sausage may contain a substantial proportion of grains, breadcrumbs and other non-meat fillers), it is also necessary to apportion the meat/non-meat components accordingly. This is achieved using reference values from McCance and Widdowson’s ‘The Composition of Foods’ [33], the same source which the main NDNS disaggregation scheme utilises [32]. As an example, McCance and Widdowson [33] estimate that premium sausages contain 77.5% meat whereas economy sausages contain 38% meat. We use the appropriate values alongside the NDNS product description and weight to estimate the quantity of meat contained in the product. If the product description suggests it contains multiple meat types (e.g. pork and beef sausage), the quantity is distributed accordingly.

### Identifying demographic groups for targeted policy intervention

The analysis in this study assumes an idealised scenario where all individuals who currently consume above the WCRF guidelines reduce their intake to meet the criteria, whilst those who already meet the criteria do not change their diets. In reality, it is likely that universal guidelines (such as those supplied by the WCRF) would result in some existing low consuming individuals reducing their intake to well below the guidelines, whilst other high consuming individuals may not adequately reduce theirs. If these groups can be identified, TPIs present a way of offering bespoke advice/incentives. Preferentially targeting high consumption groups will maximise resources and reduce the risk of negative trade-offs. There would be minimal benefit (in terms of reducing bowel cancer prevalence) of targeting groups where red and processed red meat is already below the WCRF guidelines. If these groups were targeted and further reduced their consumption they may be at increased risk of secondary negative impacts such as nutrient deficiency.

To explore the potential of a targeted approach in relation to the WCRF guidelines, we used NDNS consumption data to identify demographic groups who currently consume above and below the guidelines. Specifically, we disaggregated red and processed red meat consumption by 16 separate age-sex groups as there is known to be a strong relationship between meat consumption and these variables [34, 35]. To best align with the WCRF guidelines, this section of analysis uses the full weight of processed red meat products (e.g. including any ’fillers’ in sausages).

### Aligning diets to WCRF recommendations

As discussed in “Background” section, the WCRF issues different guidelines for processed red meat (‘very little, if any’) and non-processed red meat (No more than 500g cooked weight per week) [11]. To assess the impact of all individuals following both these recommendations as closely as possible, we modified the individual level diary data by firstly removing all products which contained processed red meat. Remaining non-processed red meat products were then iteratively randomly removed until the individual consumption was below the equivalent of 500g per week. This procedure was performed for each of the 5,000+ individuals in our survey dataset, and then repeated 100 times to analyse the sensitivity of product removal order. For each product removed, the associated non-meat components of the product were also removed. For example, if a sausage roll was identified for removal, both the sausage and pastry ingredients were removed. This is partly because the NDNS provides nutrient data at the entire product level (as used in “Methods for assessing nutrient intake” section), but also because it results in a more realistic modification to the food diary. For example, it would not be expected for an individual to consume solely the pastry from a sausage roll if the meat portion was removed.

Although there is no universally accepted definition of ‘processed’ red meat, the WCRF states that the term refers to meat which has been smoked, cured or had salt or chemical preservatives added. To best follow this definition, and in accordance with other studies (e.g. [13, 36],), we classify sausages and burgers as processed meat products. Unprocessed red meat refers to all types of meat from mammals, such as beef, veal, pork, lamb, mutton, horse and goat. As such, we used the NDNS disaggregated categories of processed red meat, sausages, burgers and grilled steaks to identify the presence and quantity of processed red meat and the categories of beef, lamb, pork, other red meat and offal for non-processed red meat.

By removing products in the manner described, a ’post-intervention’ food diary is generated for each individual, consisting of all the remaining food items. This is compared to the original food diary (termed ’pre-intervention’) to investigate changes in nutrient intake, total consumption and global warming potential.

### Methods for assessing nutrient intake

To investigate the impacts of adhering to the WCRF guidelines on nutrient intake, we compared pre-intervention estimates of iron, zinc, vitamin B12, vitamin B3, fat and saturated fat with those achieved post-intervention. We disaggregated nutrient intake by age-sex and by socio-economic classification in the form of the National Statistics Socio-economic Classification (NS-SEC) [37] for each individual. NS-SEC divides the population in to eight groups from the most affluent (Higher managerial and administrative) to the least affluent (Never worked and long-term unemployed). For both the pre- and post-intervention diets we do not include nutrients derived from supplements (e.g. vitamin tablets). The nutrients chosen are abundant in red and processed red meat and the NDNS provides data on their prevalence in each food product. These nutrients examined by no means form an exhaustive list of those impacted by adhering to the WCRF guidelines, but were chosen to highlight the variety of impacts possible and to demonstrate the techniques applied. Whilst salt (sodium) is a nutrient abundant in processed red meat products [3], it was not analysed due to lack of appropriate data. Specifically, the NDNS food diary does not include salt from discretionary sources (e.g. added during cooking or at the table) due to limitations in self reporting and quantification [38]. The NDNS does assess overall sodium intake via a separate urinary sodium survey [38], but these data are incompatible with our analysis as they do not provide information on the consumption source. For nutrients where deficiency is the likely public health concern (e.g. iron, zinc, vitamin B12, vitamin B3) the NDNS also includes estimates of Lower Reference Nutrient Intakes (LRNI’s) for each individual based on their age and sex. LRNI’s are a commonly used indicator of whether an individual is receiving sufficient intake (e.g. [30],). The LRNI represents the quantity of a nutrient that is likely to meet the needs of only 2.5% of the population group [39]. This means that in a normal population group, 2.5% of individuals would be expected to have requirements below the LRNI. Intakes below this level are almost certainly inadequate. We compared pre- and post-intervention nutrient intakes (in terms of proportion of LRNI met) to assess the impact of adhering to the WCRF guidelines. Of particular interest are cases where individuals originally received sufficient intake (above LRNI) but fell below the recommended levels (below LRNI) post-intervention.

Whilst fat is a major contributor of energy intake, there are no deficiency signs that are specifically associated with inadequate intake and therefore LRNIs are inappropriate [39]. Over-consumption of fat is the primary health concern (especially in developed countries) due to risk factors associated with many chronic diseases such as diabetes and cancer [40, 41]. Saturated fats are especially known to increase the risk of cardiovascular disease and coronary heart disease [42]. Recommended maximum fat intakes (for both total fat and saturated fat) are therefore commonly expressed in terms of percentage of total energy intake [43]. The Department of Health and Social Care recommends that no more than 33% of total energy should be derived from fats and no more than 10% should be derived from saturated fats [39]. We compared pre- and post-intervention fat intakes (both total and saturated) to these values to assess the impact of adhering to the WCRF guidelines.

### Methods for assessing total consumption

We estimated overall consumption of each meat type for both the pre- and post-intervention scenarios. We achieved this by multiplying per-capita consumption figures by total population figures [44]. Both consumption and population estimates were stratified by 16 age-sex groups to account for demographic variations known to occur in meat consumption (e.g. [35, 45],). Age group boundaries were based on those commonly used for dietary and nutrient analysis [46]. In addition to contemporary estimates, we also projected consumption estimates to the year 2050 using official population projections [47]. We used the principal, low and high population projection variants (stratified by age and sex) to capture a range of population growth/demographic change scenarios.

Consumption estimates were generated solely for the meat content of products using the steps outlined in “Non-meat content in foods” section (e.g. we do not include ’filler’ ingredients of a sausage). This is important for enabling robust comparisons between meat types and has previously been highlighted as an issue when dealing with meat consumption statistics [31]. Our analysis is focused on the cooked weight of consumed meat as dictated by the NDNS surveying protocols and matching WCRF guidelines. Therefore what we are measuring is the direct impact of pre- and post-intervention consumption, taking in to account potential future population change. This is in contrast to consumption estimates by the Organisation for Economic Co-operation and Development (OECD) and Agriculture and Horticulture Development Board (AHDB) which report consumption in terms of carcase weight [48–51].

### Methods for assessing greenhouse gas emissions

Global warming potential (GWP), expressed in terms of kg CO_2_-eq is the standard method for comparing the climate effects of emissions of different greenhouse gases [52]. We estimated the GWP of meat consumed in the UK by combining our consumption estimates (“Methods for assessing total consumption” section) with GWP coefficients provided by Clune et al. [53]. In line with the NDNS, the domain of our GWP analysis covers meat consumed in the UK regardless of production location. However it does not include any meat produced in the UK exported overseas, nor does it include meat not used for human consumption (e.g. pet food) or any wastage. Following the scope of this paper, the aim of the GWP analysis is to assess the potential impacts of the WCRF guidelines within the described domain, rather than to produce a comprehensive estimate for the entire industry.

The original database of GWP coefficients produced by Clune et al. [53] is based on a meta-analysis of 369 peer reviewed studies and provides coefficients in terms of a common functional unit and system boundary of kg CO_2_-eq/kg bone free meat at the regional distribution centre. Separate coefficients are provided for each meat type (beef, lamb, pork) and for different regions of production. As meat consumed in the UK originates from a variety of geographical locations, we firstly apportioned our consumption estimates to the regions used by Clune et al. [53]. The proportion of UK meat consumption derived from UK production was provided by the AHDB who publish figures detailing ‘domestically used production as a % of consumption’ [48–50]. For the remainder, we used import data [54–56] to calculate the relative contribution from regions outside the UK. For the small proportion of imports whose origin was listed as ‘other’, we assigned the Clune et al. [53] world average coefficient for the appropriate meat type shown in Table 1.

**Table 1:** Regional source of UK meat consumption and associated GWP (kg CO_2_-equivalent)

Meat Type	Region	% UK Consumption	GWP to regional distribution centre	GWP Travel to UK	Total GWP
Pork	UK	38.3	6.11	0.00	6.11
Pork	EU	61.6	5.39	0.03	5.42
Pork	North America	0.05	6.00	0.05	6.05
Pork	Other	0.05	5.74	0.07	5.81
Beef	UK	63.13	26.57	0.00	26.57
Beef	EU	34.75	24.96	0.03	24.99
Beef	South America	0.95	34.10	0.05	34.15
Beef	Australia	0.34	22.88	0.11	22.99
Beef	Other	0.83	26.61	0.07	26.68
Lamb	UK	68.66	24.48	0.00	24.48
Lamb	EU	5.95	32.70	0.03	32.73
Lamb	Australia & New Zealand	24.46	17.63	0.12	17.75
Lamb	Other	0.94	25.58	0.07	25.65

Due to the global scope of Clune et al. [53], their GWP coefficients only account for emissions up to the regional distribution centre. In our study, additional transport emissions (‘food miles’) will be present when the regional distribution centre is situated outside the UK (e.g. lamb produced in New Zealand and consumed in the UK). These emissions need to be considered when analysing UK consumption from different production regions. To achieve this, we applied transport emission estimates in a similar approach to Saunders & Barber [57]. For each geographical region, we estimated the distance to the UK (for the EU we used a distance of 1,000 km to approximate the central point). For overseas regions we applied the shipping emission coefficient of 0.007 kg CO_2_ per tonne km [58] and for the EU we used the road (truck) emission coefficient of 0.027 kg CO_2_ per tonne km [58]. No additional emissions were added for meat produced in the UK.

By multiplying our consumption estimates (disaggregated by meat type and production location) by the corresponding GWP coefficient (including transport emissions to the regional distribution centre) we were able to produce bulk GWP estimates for the domain described. As with our consumption estimates (“Methods for assessing total consumption” section), we generated GWP estimates for both original and post-intervention diets, projected to the year 2050.

## Results

### Identifying demographic groups for targeted policy intervention

**Fig. 1:**
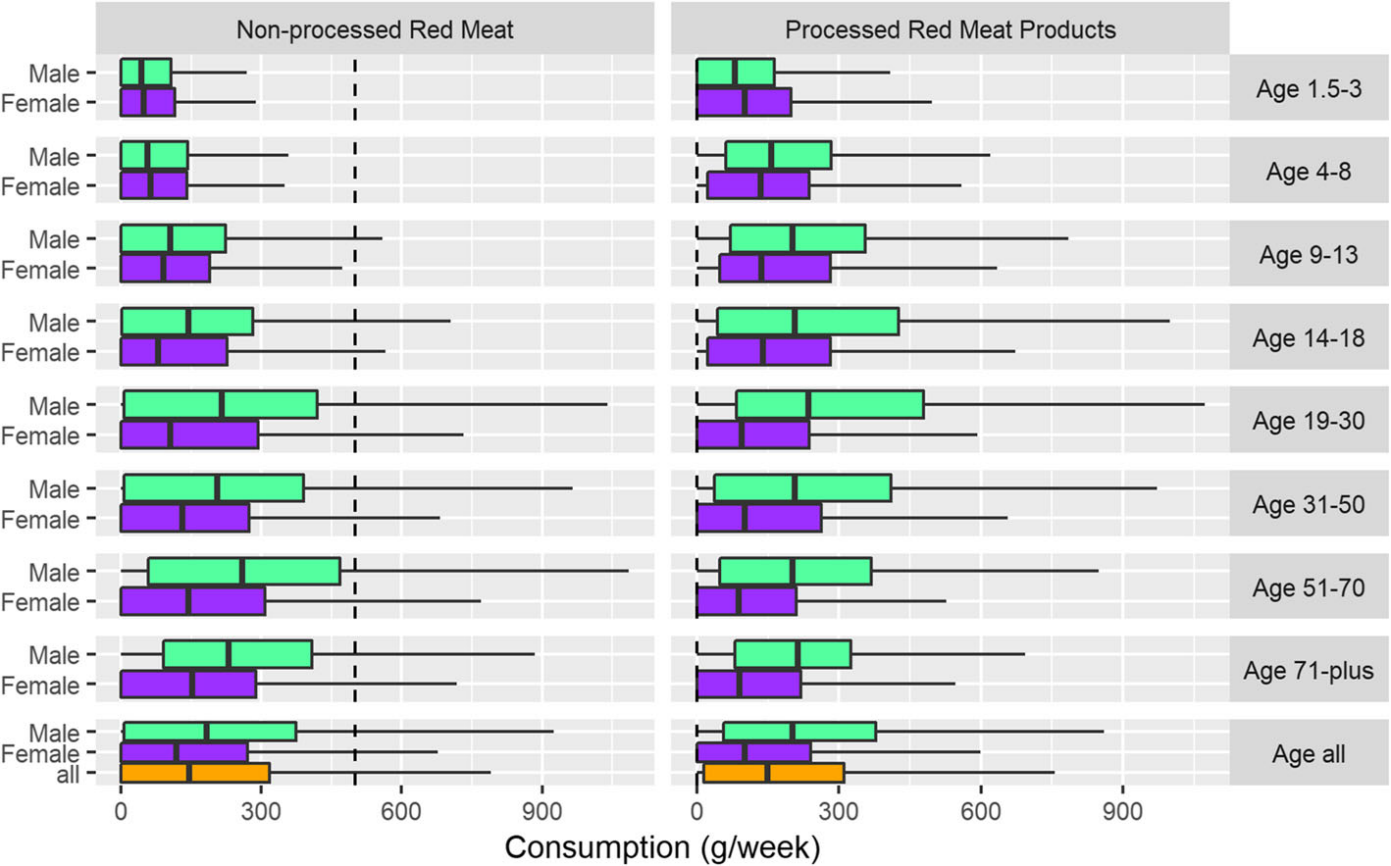
Boxplots showing processed and non-processed red meat consumption in relation to WCRF guidelines. Dashed lines indicate WCRF recommendations

Table 2 and Fig. 1 shows how consumption varies by age-sex groups. Across the entire population, pre-intervention median weekly consumption of non-processed red meat was 145.3g and 148.9g for processed red meat, with 11.1% and 76.7% of the population exceeding the WCRF guidelines respectively. The high proportion of individuals exceeding the processed red meat guidelines is to be expected due to the threshold of zero (i.e. only those recording no processed red meat consumption whatsoever would meet the requirements). Of interest to this study is the substantial variation in both processed and non-processed red meat consumption by age-sex groups. Median consumption (all age groups) for males was higher than for females for both non-processed red meat (182.1g vs 117.4g per week) and processed red meat (201.2g vs 101.2g per week). This is reflected in the proportion of males and females exceeding the WCRF guidelines for non-processed (14.5% vs 7.8%) and processed (80.9% vs 72.5%). There was also substantial variation by age groups. For example, 85.8% of males aged 71 plus exceeded the processed threshold compared to 67.1% of females aged 51-70 years.

**Table 2:** Processed and non-processed red meat consumption (weekly) and proportion of individuals exceeding WCRF guidelines

Age (years)	Sex	Median consumption (g): non-processed	% above WCRF: non-processed	Median consumption (g): processed	% above WCRF: processed
1.5-3	Male	42.7	1.5	80.5	72.3
1.5-3	Female	48.2	0.3	101.6	72.9
4-8	Male	56.8	1.0	157.4	82.0
4-8	Female	62.0	1.4	134.8	79.4
9-13	Male	104.8	1.9	202.2	84.2
9-13	Female	89.6	3.2	135.7	81.9
14-18	Male	143.1	7.9	207.4	79.1
14-18	Female	78.8	5.5	139.3	79.1
19-30	Male	215.5	17.8	235.7	84.0
19-30	Female	105.3	10.2	94.1	71.7
31-50	Male	205.0	15.9	206.6	79.0
31-50	Female	130.3	8.0	101.6	67.1
51-70	Male	259.1	21.7	201.2	79.6
51-70	Female	143.5	8.4	87.5	72.8
71-plus	Male	230.4	12.6	213.9	85.8
71-plus	Female	152.1	10.9	89.7	74.4
all	Male	182.1	14.5	201.2	80.9
all	Female	117.4	7.8	101.2	72.5
all	all	145.3	11.1	148.9	76.7

### Nutrient intake results

**Fig. 2:**
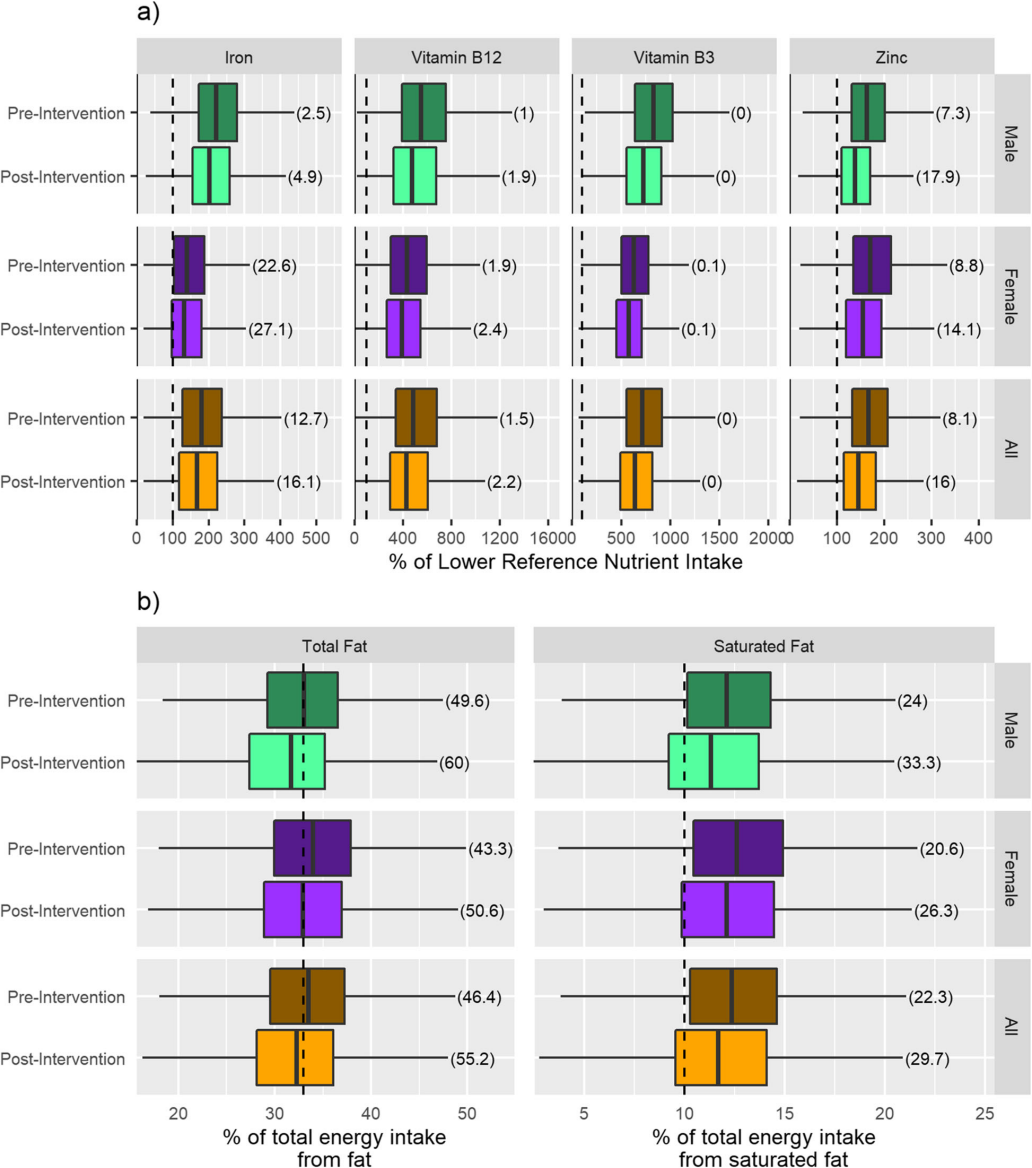
Boxplots showing pre- and post intervention nutrient intakes (all age groups). **a** Nutrients with a recommended minimum intake (LRNI). Numbers in brackets indicate % of population below LRNI. Dashed lines indicates 100% of LRNI/ **b** Nutrients with a recommended maximum intake. Numbers in brackets indicate % of population below recommended limit. Dashed lines indicate recommended maximum intake

Pre- and post-intervention intakes of iron, zinc, vitamin B12, vitamin B3, total fat and saturated fat are shown in Fig. 2 and Table 3. Intakes are reported in terms of % of LRNI met or % of total energy intake, as described in “Methods for assessing nutrient intake” section. Figure 2 shows the results for the entire population whilst Table 3 shows results stratified by sex and age-sex groups. In common with previous studies (e.g. [15]) there was large variability in pre-intervention nutrient intake. For example, when considering all age-sex groups combined (Fig. 2), 12.7% of individuals had an iron intake below the LRNI, compared to just 0.03% for vitamin B3. There was also substantial variability across age-sex groups, with 55.84% of females aged 14-18 having an iron intake below the LRNI, compared to just 0.66% of males aged 4-8.

**Table 3:** Pre- and post-intervention nutrient intakes for all age groups. Fat and saturated fat in terms of % of population below maximum recommended limits (33% and 10% of total energy respectively), all other nutrients in terms of % of population below LRNI

		% of population below LRNI								% of population below limit			
Age	Sex	Vitamin B12		Iron		Zinc		Vitamin B3		Fat		Saturated Fat	
		Pre-	Post-	Pre-	Post-	Pre-	Post-	Pre-	Post-	Pre-	Post-	Pre-	Post-
1.5-3	M	0.0	0.0	8.1	11.4	5.5	13.4	0.0	0.0	42.4	49.7	11.5	14.0
1.5-3	F	0.0	0.1	11.0	16.1	6.5	14.5	0.0	0.0	36.4	47.1	4.0	9.8
4-8	M	0.0	0.0	0.7	1.8	9.2	19.2	0.0	0.0	45.4	56.8	11.9	17.8
4-8	F	0.2	0.2	1.7	3.5	14.9	26.9	0.0	0.0	42.6	55.8	9.6	13.2
9-13	M	0.0	0.0	4.1	8.4	10.9	22.7	0.0	0.0	44.2	60.2	11.4	18.7
9-13	F	1.6	2.4	27.0	33.3	24.4	34.2	0.0	0.0	41.6	51.9	18.3	25.0
14-18	M	0.5	3.3	11.8	17.9	18.0	28.8	0.0	0.0	47.6	60.0	21.2	30.9
14-18	F	2.3	3.5	55.8	61.0	17.7	30.6	0.0	0.0	42.7	53.7	17.5	26.9
19-30	M	2.3	5.1	1.1	5.3	6.9	24.7	0.0	0.0	54.6	70.0	29.7	43.9
19-30	F	2.8	3.1	41.0	47.7	6.0	10.7	0.0	0.0	45.3	52.8	24.3	32.5
31-50	M	0.8	1.4	1.7	2.6	5.5	14.9	0.0	0.0	47.4	56.3	27.8	38.2
31-50	F	2.7	3.9	31.2	38.4	9.0	12.2	0.0	0.0	45.5	51.6	26.2	33.5
51-70	M	0.8	1.4	1.6	3.9	5.4	13.9	0.0	0.0	54.1	62.9	26.6	34.3
51-70	F	1.8	1.9	6.9	8.2	4.9	8.6	0.2	0.2	44.3	50.2	23.4	26.6
71+	M	1.8	1.8	1.7	2.1	8.6	15.9	0.0	0.0	46.5	52.9	19.4	24.9
71+	F	0.3	0.3	6.2	8.6	6.2	10.7	0.0	0.0	36.4	43.0	9.0	11.4
All	M	1.0	1.9	2.5	4.9	7.3	17.9	0.0	0.0	49.6	60.0	24.0	33.3
All	F	1.9	2.4	22.6	27.1	8.8	14.1	0.1	0.1	43.3	50.6	20.6	26.3
All	All	1.5	2.2	12.7	16.1	8.1	16.0	0.0	0.0	46.4	55.2	22.3	29.7

As expected, post-intervention nutrient intakes were lower than pre-intervention for all nutrients. For nutrients of deficiency concern (iron, zinc, vitamin B12, vitamin B3), this routinely resulted in an increased proportion of individuals receiving intakes below the LRNI. For these nutrients, the largest changes at the population level were evident for zinc, where the proportion of individuals not meeting the LRNI increased from 8.07% to 15.96% (+7.89%) and for iron, where the increase was from 12.72% to 16.15% (+3.43%). In contrast, the proportion of individuals not meeting the LRNI for Vitamin B12 increased by just 0.76% (1.46% to 2.22%) whilst for vitamin B3 intake remained unchanged, with just 0.03% of the population failing to meet the LRNI. For each nutrient, there was also significant variation in the impact of intervention by age-sex group (Table 3), a fact which is not evident when considering the entire population data alone. For example, at one end of the spectrum, the proportion of males aged 19-30 not meeting the LRNI for zinc increased from 6.92% to 24.68% (+17.8%) whilst at the other, females aged 31-50 the increase was just 3.15% (from 9.01% to 12.16%). After intervention, 60.98% of females aged 14-18 failed to meet the LRNI for iron, the highest value for any age-sex group across all nutrients.

For nutrients with an over-consumption concern (total fat, saturated fat), adherence to the WCRF guidelines resulted in an increased proportion of individuals meeting the recommended limits. At the population level, the % of individuals meeting the recommended limit for total fat rose from 46.4% to 55.2%. For saturated fat, the proportion of individuals meeting the recommended limit rose from 22.3% to 29.7%. There was also significant variation in the impact of intervention by age-sex group (Table 3). For example, the proportion of males aged 9-13 meeting the recommended limits fro fat intake increased from 44.2% to 60.2% (+16.0%) whereas for females aged 51-70 the increase was just 5.9% (44.3% to 50.2%).

**Fig. 3:**
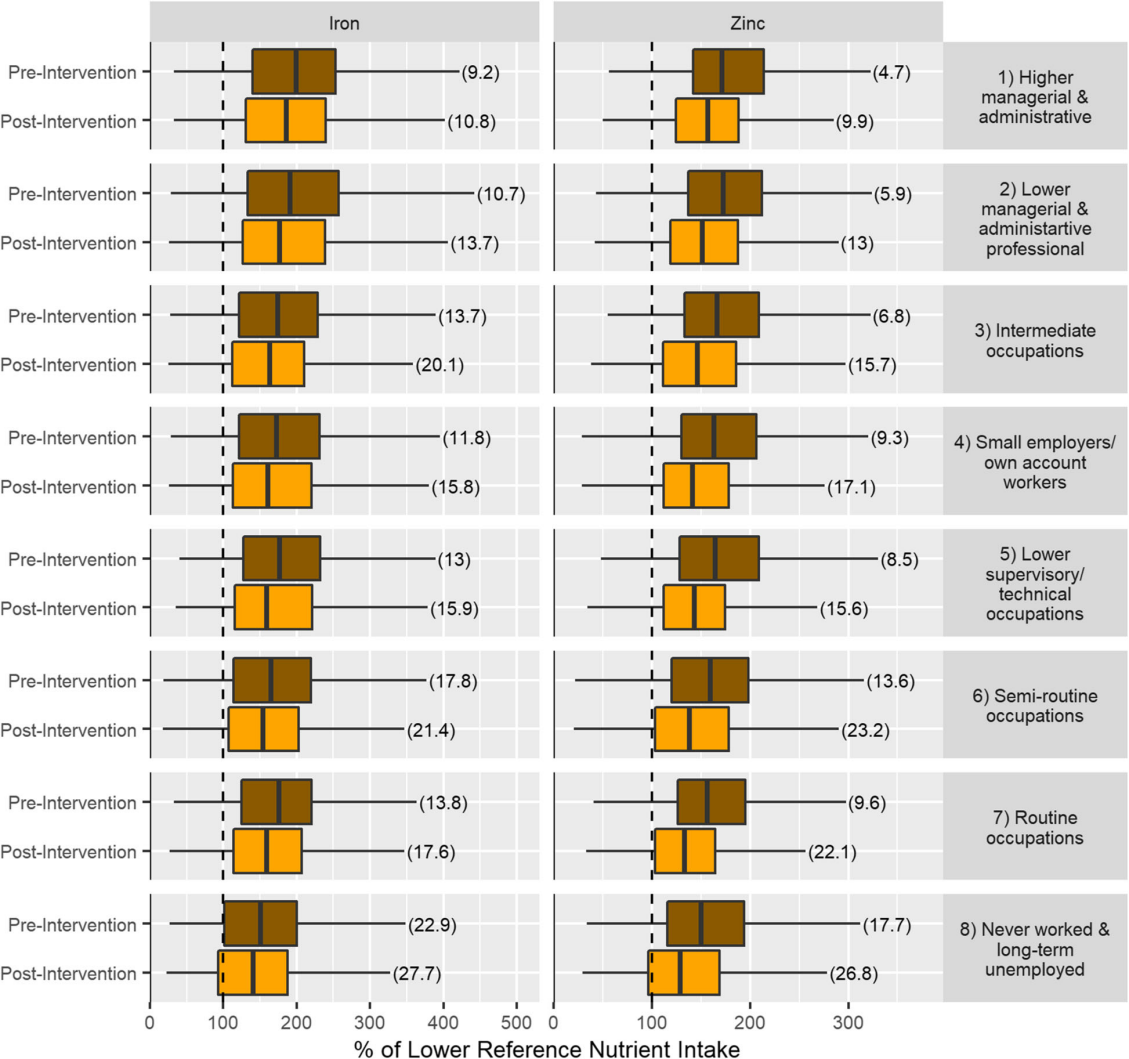
Boxplots showing pre- and post intervention iron and zinc intakes based on NS-SEC classification (of each individuals corresponding household reference person). Numbers in brackets indicate % of population below LRNI. Dashed lines indicates 100% of LRNI

Figure 2 and Table 3 shows that iron and zinc exhibit substantial deficiency concerns for both specific age-sex groups and at the population level even before intervention. We therefore performed additional analysis on these nutrients based on disaggregation by NS-SEC categories [37] to explore the impact upon different socio-economic groups. Figure 3 shows that pre-intervention, NS-SEC group 1 (Higher managerial and administrative) exhibited the lowest proportion of individuals failing to meet the LRNI for iron and zinc (9.2% and 4.7% respectively). Similarly, post intervention proportions were also lowest for NS-SEC group 1 (10.8% and 9.9% respectively). In contrast, NS-SEC group 8 (Never working and long-term unemployed) exhibited the highest proportion of individuals failing to meet the LRNI for iron and zinc both pre-intervention (22.9% and 17.7%) and post intervention (22.7% and 26.8%).

### Total consumption results

**Fig. 4:**
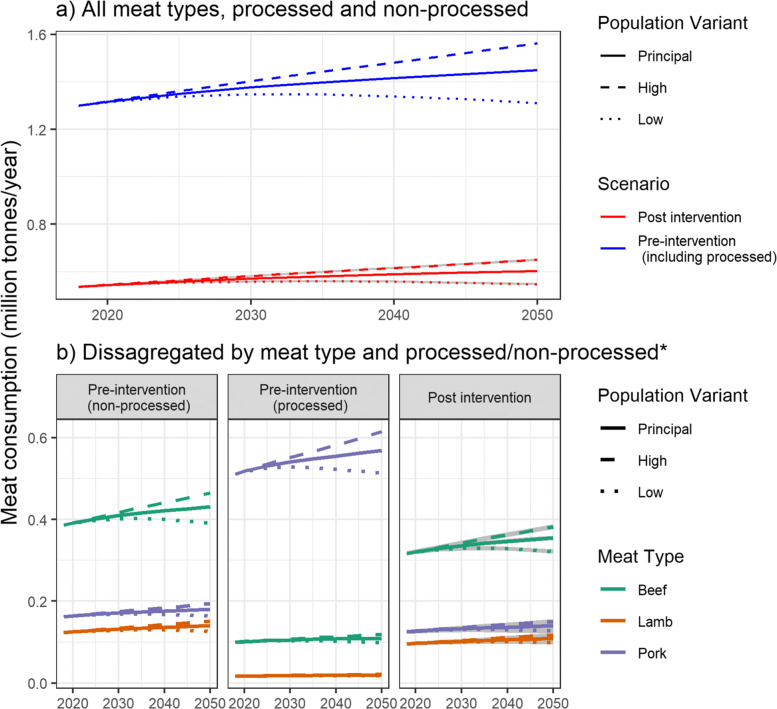
Projections of UK red and processed red meat consumption. Scenarios of demographic change only (pre intervention) and including per-capita changes following alignment to WCRF guidelines (post intervention). Grey shading indicates sensitivity to product removal order. **a** aggregated consumption from all meat types. **b** consumption disaggregated by meat type and processed/non processed. *no disaggregation for processed/non processed post innervation as all processed products removed

For the year 2018 and under the pre-intervention scenario, total consumption for all processed and unprocessed red meat was 1.30 million tonnes per year (Fig. 4). By the year 2050 and under a continued pre-intervention scenario, this is projected to increase to 1.45 million tonnes (+11.5%) using the principal population projection variant, 1.31 million tonnes (+0.9%) using the low population projection variant and 1.56 million tonnes using the high population projection variant (+20.3%).

Post-intervention consumption is considerably lower (Fig. 4), with 2018 consumption just 0.54 million tonnes, 58.6% less than the pre-intervention scenario for the same year. Under the post-intervention scenario, consumption for the year 2050 is projected to be 0.60 million tonnes using the principal population projection. Using the low and high population projection variants result in 2050 consumption projections of 0.55 and 0.65 million tonnes respectively.

Figure 4 shows consumption projections disaggregated by meat type (beef, lamb, pork) and also by processed and non-processed products. As adherence to the WCRF guidelines results in zero processed red meat consumption, there is no disaggregation between processed/non-processed for the post intervention scenario. Under the pre-intervention scenario, beef accounted for the majority of non-processed meat consumption (57.5%; 0.39 million tonnes in 2018), with pork and lamb accounting for 24.1%; 0.16 million tonnes and 18.4%; 0.12 million tonnes respectively. This is in contrast to processed meat consumption (Fig. 4) where pork accounted for 83.7%; 0.50 million tonnes, beef 15.4%; 0.09 million tonnes and lamb 0.9%; 0.01 million tonnes.

The post intervention scenario shows a much larger decrease for pork consumption compared to other meats. Specifically, pork consumption for the year 2018 fell from 0.66 to 0.13 million tonnes, a reduction of 81.4%. For comparison, beef consumption reduced from 0.49 to 0.32 million tonnes (-34.9%) whilst lamb fell from 0.14 to 0.10 million tonnes (-31.9%).

### Greenhouse gas emission results

**Fig. 5:**
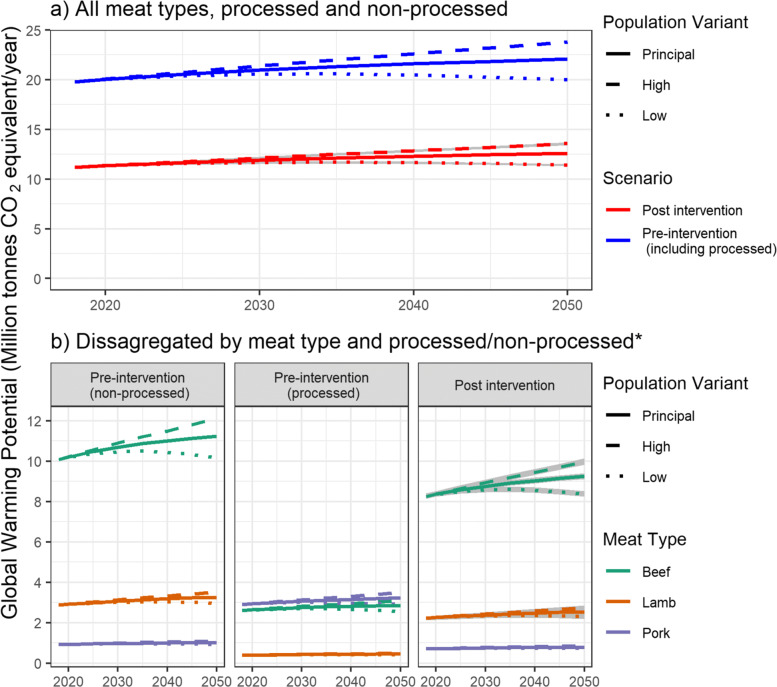
GWP projections from UK red and processed red meat consumption. Scenarios of demographic change only (pre intervention) and including per-capita changes following alignment to WCRF guidelines (post intervention). Grey shading indicates sensitivity to product removal order. **a** aggregated emissions from all meat types. **b** emissions disaggregated by meat type and processed/non processed. *no disaggregation for processed/non processed post innervation as all processed products removed

For the year 2018 and under the pre-intervention scenario, total GWP from all processed and non-processed red meat is estimated to be 19.77 million tonnes CO_2_ eq per year (Fig. 5). Under a continued pre-intervention scenario and assuming the principal population projection, this is projected to increase by 11.6% to 22.06 million tonnes CO_2_ eq by 2050. For comparison, the low population projection variant results in an increase of 1.0% whereas the high population variant results in an increase of 20.3%. GWP is lower under the post-intervention scenario(s) (Fig. 5), with 2018 emissions estimated to be 11.2 million tonnes CO_2_ eq per year, 43.4% lower than pre-intervention. Under the post-intervention scenario, GWP for the year 2050 is projected to be 12.6 million tonnes CO2 eq per year using the principal population projection. The low and high population projection variants result in 2050 GWP projections of 11.4 and 13.6 million tonnes CO_2_ eq per year respectively.

Pre-intervention, beef accounted for 72.7% of the GWP from non-processed meat consumption, with pork and lamb accounting for 6.6% and 20.7% respectively (Fig. 5). This is in contrast to processed meat consumption, where pork accounted for 49.0%, beef 44.2% and lamb 6.7%. As with consumption, post-intervention scenarios resulted in a much larger decrease in GWP from pork compared to other meats. Specifically, pork GWP (for 2018) fell from 3.8 to 0.7 million tonnes CO2 eq per year (81.1% reduction). For comparison, beef consumption reduced from 12.7 to 8.3 million tonnes (33.9% reduction) whilst lamb fell from 3.3 to 2.2 million tonnes (25.6% reduction).

## Discussion

Our post intervention scenarios demonstrate there would be a range of trade-offs, conflicts and secondary impacts, even if a targeted policy intervention strategy was fully successful (i.e. all individuals who currently consume above the WCRF guidelines reduce their intake to meet the criteria, whilst those who already meet the criteria do not change their diets).

Analysis of nutrient intake data (“Nutrient intake results” section, Fig. 2 and Table 3) shows that positive health benefits may be realised by reduced intake of some nutrients (e.g. total fat and saturated fat). However, negative health impacts may also occur as the proportion of individuals failing to meet the LRNI for a range of nutrients (e.g. iron, zinc, vitamin B12) increases under the post-intervention scenario. This shows that policies directed at reducing meat consumption need to consider the composition of substitute foods in order to mitigate the reduction of certain nutrients whilst preserving the positive health impacts. Of the nutrients analysed, zinc and iron are especially of note, with the proportion of individuals failing to meet their LRNI increasing by 7.89% and 3.43% respectively. For each specific nutrient there is also substantial variability between demographic groups. For example, the proportion of males aged 19-30 not meeting the LRNI for zinc increased by 17.9% whilst for females aged 31-50 the increase was just 3.15%. This suggests that a further level of targeted policy intervention may be beneficial to advocate the most appropriate substitute products for specific demographic groups (e.g. encourage males aged to 19-30 to substitute meat products with alternatives containing a high zinc content such as oysters). Special attention should also be paid to iron intake and women of childbearing age, with our analysis showing the percentage of women aged 19 to 30 not achieving the LRNI for iron rose from 40.1% pre intervention to 47.7% post-intervention. This is especially a concern as iron deficiency in pregnancy is a risk factor for preterm delivery and subsequent low birth weight, and possibly for inferior neonatal health [16]. Whilst this is already a recognised issue (with supplementary iron often prescribed during pregnancy), the prevalence is likely to increase under a backdrop of dietary change. Analysis of nutrient intake by NS-SEC (Fig. 3) demonstrates how impacts of the intervention might be differently felt by socio-economic sub-group. Notably the proportion of people in the Never worked and long-term unemployed group with iron and zinc intake below recommended levels is high pre intervention (22.9% and 17.7% respectively), rising substantially post-intervention to 27.7% and 26.8%. This suggests that any policy to reduce red meat consumption would need to pay particular attention to less affluent groups in order to ensure there were not detrimental nutritional impacts.

Our analysis of overall meat consumption (“Total consumption results” section and Fig. 4) demonstrates that adherence to WCRF guidelines would have a major impact, reducing overall consumption by 58.6%. Whilst future consumption levels may increase slightly due to population growth, this would be negligible compared to the per-capita losses due to adherence to WCRF guidelines, even assuming a high population growth rate scenario (Fig. 4). Reduced consumption would invariably result in lower demand for UK retail, packaging and distribution networks by approximately the same amount. Impacts on UK based production are more complex as demand relies on consumption both within the UK and from exports. As our analysis only covers UK based consumption, our results are only applicable for the portion of production sourced from the UK. Table 1 shows that UK produced lamb currently accounts for 67% of UK lamb consumption, whilst the contribution for beef and pork is 63% and 38% respectively. This suggests that the UK lamb and beef industries are more sensitive to changes in domestic consumption than pork which is more reliant on imports.

The potential of unemployment in the sector is a concern, not least because of detrimental social and health impacts this can have on individuals [59, 60]. To avoid net employment loss, any job losses in the meat industry would need to be replaced by a similar number of equivalent jobs in other industries. The impacts of sudden mass unemployment in a sector were seen during the decline of coal mining in the UK, where employment fell from 240,000 in 1981 to just 6,000 by 2011 [61]. Regions which experienced widespread mine closure were still suffering from unemployment and deprivation over 20 years later [62], highlighting the potential risks of widespread change in a particular industry and the need for relevant policy to minimise negative impacts.

Disaggregating consumption estimates by meat type (Fig. 4) reveals that post-intervention scenarios have a disproportionate impact on pork consumption compared to beef and lamb. Specifically (for the year 2018), pork consumption is reduced by 83.7% compared to 34.9% for beef and 31.9% for lamb. This is due to the prevalence of processed pork products (compared to beef or lamb) and the much stricter WCRF guidance for processed products (zero consumption), compared to non-processed products (no more than 500g/week). There is no evidence that the variation of processed/non-processed products by meat type has previously been discussed when addressing dietary guidance, showing how a technical detail in guidance/policy can have a significant impact on final outcomes. This phenomenon would not occur if processed and non-processed products were treated equally in the original policy guidance, showing the importance of carefully considering how policies are designed.

As expected, reduced meat consumption due to alignment to the WCRF guidelines would result in reduced GWP. Figure 5 shows that post-intervention scenarios reduce overall GWP by 43.4%. Importantly, these figures do not account for the emissions generated from any substitute products and it is therefore expected that the net GWP reductions would be substantially less. Despite this, alignment to WCRF guidelines is still expected to reduce net GWP as meat substitute products invariably have a lower GWP coefficient [53], although exceptions do apply. As noted by Saunders & Barber [57] and Weber & Matthews [63], transport emissions between the production location and regional distribution centre (‘food miles’) are negligible compared to total emissions (Table 1). Even for lamb produced in New Zealand (and exported to the UK), transport emissions only account for 0.68% of total emissions. This suggests that policies for reducing GWP from the meat sector should focus on the production phase rather than transport emissions, as advocated by Weber & Matthews [63]. It should be noted that this analysis does not consider other differences between meat production locations. For example, whilst meat produced in the UK has a relatively high GWP (Table 1), it has some of the best farm animal welfare in the world [64].

For each meat type, total GWP is a product of the total consumption and the appropriate GWP coefficient. Table 1 shows that beef and lamb have a much higher GWP coefficient compared to pork, as outlined by Clune et al. [53]. This is mainly because ruminants (i.e. cows and sheep) digest food through the process of enteric fermentation in a multichambered stomach, producing methane (a powerful greenhouse gas) as a by-product. Non-ruminants or ’monogastric’ animals such as pigs have a single chambered stomach to digest food, and their methane emissions are small in comparison [27]. As such, policies designed to tackle greenhouse gas emissions often focus mainly on ruminants due to their high GWP coefficient (e.g. [2]). In contrast, the WCRF guidelines (designed for bowel cancer risk reduction) result in a disproportionate reduction of pork due to the high prevalence of processed pork products (as described above). Specifically (for the year 2018), pork consumption is reduced by 83.7% compared to 34.9% for beef and 31.9% for lamb (Fig. 4). This results in smaller GWP reductions than would be achieved if consumption of all meats types was reduced equally (and even less than if beef and lamb consumption was prioritised). Whereas the post-intervention scenario (across all meat types) results in a consumption reduction of 58.6%, the comparative GWP reduction is just 43.4%. This again shows how a seemingly small nuance in guidance/policy can have a significant impact on final outcomes.

### Under-reporting of food diary data

The NDNS provides the best available data related to dietary composition in the UK and is regarded as one of the most comprehensive food diary surveys in Europe [65]. Despite this, under-reporting is inherent in food diary studies such as the NDNS and should be acknowledged as a limitation [66, 67]. Whilst methods such as doubly labelled water (e.g. [68]), Goldberg cut-offs (e.g. [69]) and estimated energy requirements (e.g. [67]) can be used to identify (and remove) under-reporters in food-diary surveys, the NDNS does not provide sufficient data to apply any of these techniques at the individual level for all respondents. Furthermore, the use of more generalised approaches (e.g. Goldberg method with a single cut-off) can lead to misclassification of a proportion of respondents [70], thus making it unsuitable for our study. Whilst the NDNS recognises under-reporting as “an area of ongoing concern and priority warranting further investigation” (e.g. [71]), the datasets are published with no attempt to quantify under-reporting at the individual level. On balance, and in line with numerous other studies (e.g. [72]), our analysis is based on the full published dataset. As such, our analysis of nutrient intake, total consumption and GHG emissions can be seen as a conservative estimate.

### Targeted policy interventions for reducing meat consumption

Previous studies have identified considerable spatial heterogeneity and inequalities in meat expenditure across demographic groups (e.g. [35, 45, 73]). This is reflected in our analysis, finding some demographic groups exceeding the WCRF consumption guidelines much more frequently than others (Fig. 1). For example, males generally consume higher quantities of both processed and non-processed red meat than women, with highest consumption in males aged 71 and over (for non-processed red meat) and males aged 19 - 30 years (for processed red meat). As such, we suggest that a targeted policy intervention strategy would be beneficial for encouraging individuals to meet the WCRF guidelines. Whilst our demographic classification is based on age and sex, it shows that adherence to WCRF guidelines varies by variables for which data is commonly available, thus offering potential for targeted policy intervention.

**Fig. 6:**
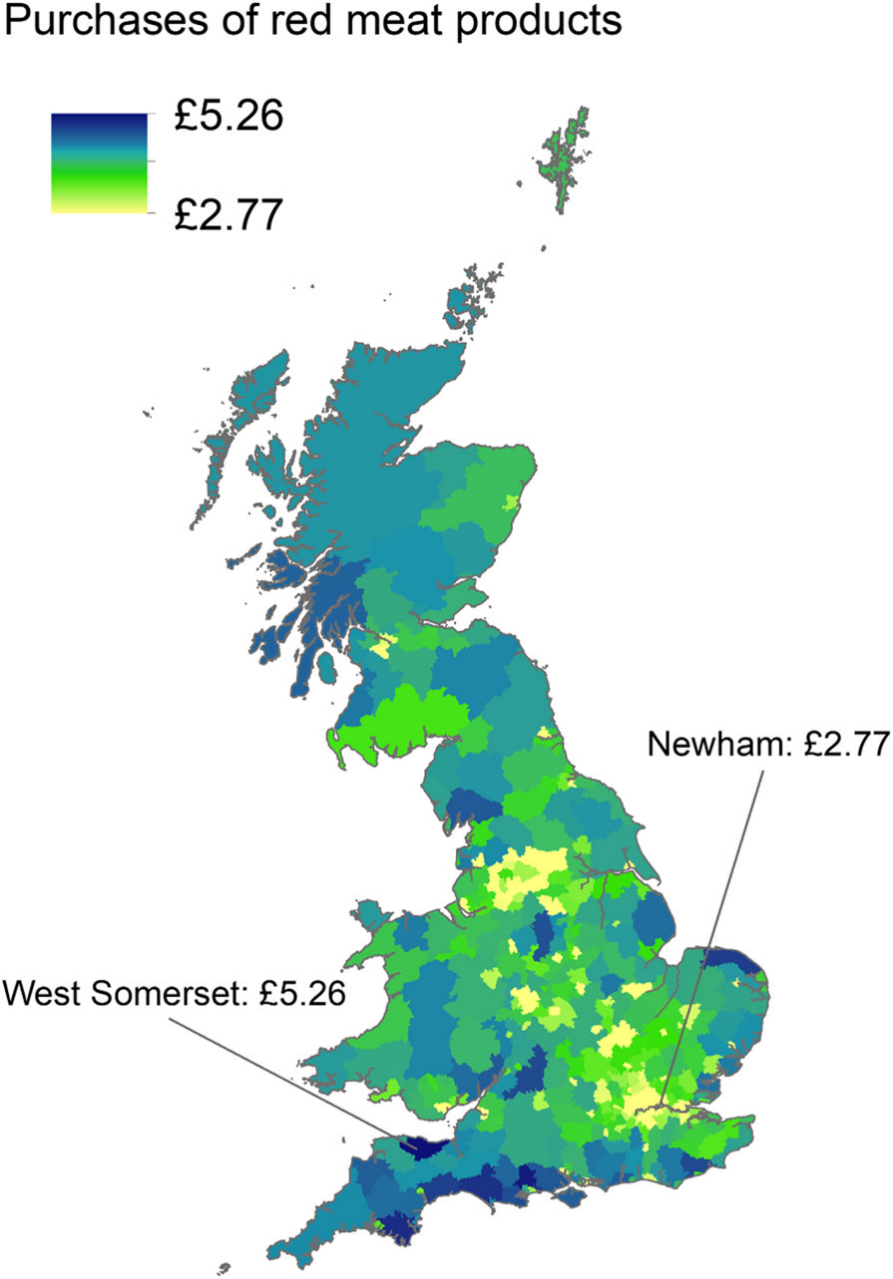
Geographical suitability for policy intervention across Great Britain based on weekly expenditure of red and processed red meat products per person (for household consumption). Purchase estimates from James et al. [45]. Boundary data source: Office for National Statistics licensed under the Open Government Licence v.3.0 Contains OS data Ⓒ Crown copyright and database right 2022. Figure generated using ArcGIS 10.6 software

Geographically targeted policy interventions could also be considered as meat consumption is known vary spatially [45, 74]. Other factors linked to changing requirements of nutrient intake (e.g. pregnancy) have considerable spatial variation [75, 76], showing how high risk geographical areas could further be identified for specific issues. Figure 6 shows how local level meat expenditure data (used as a proxy for consumption) may be used to identify regions for geographically targeted policy intervention. High expenditure regions such as West Somerset (£5.26 per person per week) would be more likely to benefit from policy interventions than low expenditure areas (e.g. Newham: £2.77 per person per week), where the risks of negative impacts would also likely be greater.

Once appropriate demographic groups/locations have been identified, there are a range of policy interventions which may be applied (Table 4). In general these can be classified into two broad categories: information measures and measures targeting the market environment. A comprehensive review by Brambila-Macias et al. [5] concluded that information measures have a mixed and limited record of success whereas measures to target the market environment are more intrusive but may be more effective. This suggests that whilst it is often logistically easier to target specific groups with information measures (e.g. the recently announced restrictions on the advertising of unhealthy foods before 9pm [7]), it is important that policies aimed at changing the market environment are also considered using a targeted approach. For example, regulation of school meals and workplace canteen meals could be focused on demographic groups/locations at higher risk.

**Table 4:** Potential policy interventions for reducing red meat consumption. Adapted from Brambila-Macias et al. [5]

Type of intervention	Further breakdown of intervention
Information Measures	
Advertising controls	On advertising to children (e.g. 9pm curfew)
	On general advertising
Public information campaigns	
Nutrition education	For children (e.g. at school)
	For adults (e.g. in workplace)
Nutritional labeling	
Nutritional information on menus	
Policies aimed at changing the market environment	
Fiscal measures	Taxes or subsidies on foods to the population at large
	Subsidies (e.g. vouchers) to disadvantaged consumers
Regulation of meals	School meals (e.g. provision of meat alternatives)
	Workplace canteen meals
Nutrition-related standards (e.g. limits on meat content and portion sizes)	Government action to encourage private sector action (e.g. reformulation)

## Conclusion

In this paper we have set out the potential impacts of adopting a specific set of dietary guidelines from the WCRF, which recommend a reduction in red and processed red meat consumption. The well documented first order objective of these recommendations is to reduce colorectal and other cancers which have been linked to consumption of these foods.

However we consider a range of conflicts, trade-offs and secondary impacts related to dietary impact (nutrient intake), greenhouse gas emissions and total consumption. By taking this broad approach we highlight that while there will always be trade offs across different areas when adopting recommendations, taking a more holistic and less siloed view can help understand what these trade-offs are. Our work serves to highlight that good information is necessary in order to assess these impacts across multiple domains and we have focused on areas where impacts can be calculated from existing information. While we work through the lens of a single scenario in the form of the WCRF recommendations, this approach can and should be taken to assess any other potential policy change. The methodology also allows any changes to be regularly reviewed in a systematic manner. This is important as dietary changes and nutrition transitions are constantly altering potential impacts [77].

While our holistic approach is a novel and powerful way of assessing impacts across the multiple domains of consumption, nutrient intake and GHG emissions, it is not without limitations. Most notably there is considerable complexity and nuance within each of the domains which require further detailed analysis to unpick. For example, we disaggregate the impact that the WCRF scenario has on nutrient intake by age, sex and socio-economic status but there are further sub-groups who may see different impacts. This would be a fruitful avenue for further investigation. We also acknowledge limitations of potential under-reporting in the NDNS data used in our analysis, which are outside of our control to consider in this paper. As we note, this means that the results reported across all domains should be seen as a conservative estimate.

The prospect of reduced meat consumption in the UK is recognised by the National Farmers Union (NFU), suggesting that “The increasing popularity of more flexitarian diets is likely to continue” [78]. The role of demographics in changing consumption patterns is also acknowledged, with an “aging consumer market” noted as one of the weaknesses of the British lamb industry [79]. Whilst our study is applicable to the UK, the global meat industry is expected to expand, with the FAO projecting worldwide meat consumption to double by 2050, mostly in developing countries, due to rising incomes and urbanisation [80]. So while the implications of adopting health guidelines such as those proposed by the WCRF would be to further reduce domestic consumption, it is important to remember that the meat industry is global and impacted by consumption behaviour elsewhere. Despite our UK focus, we find that the reduction in global warming potential is substantial when implementing the WCRF recommendations for domestic consumers. There are also substantial implications for nutrient intake once the recommendations are implemented within our model which are apparent for different demographic groups.

A targeted policy intervention strategy would be the most effective way to realise the benefits of the guidelines proposed by the WCRF whilst minimising secondary negative impacts. We shows that demographic groups for policy intervention can be identified using readily available and reliable data (e.g. age-sex), although alternative classification methods (e.g. NS-SEC) are also appropriate if the required data exists. Adherence to WCRF guidelines varies considerably by both demographic variables and spatially across the UK, providing a robust methodology for identifying target groups. Whilst ’information based’ targeted policy intervention strategies for improving diets are already used/proposed in the UK (e.g. [7]), it is likely that stronger policies aimed at changing the market environment will be needed to have a major impact on meat consumption.

## Data Availability

The derived datasets generated during the current study are available from the corresponding author on reasonable request. The raw data from the National Diet and Nutrition Survey are available from the UK Data Service (https://ukdataservice.ac.uk) but restrictions apply to the availability of these data, which were used under license for the current study, and so are not publicly available. Data are however available from the authors upon reasonable request and with permission of the UK Data Service.

## Abbreviations

AHDB: Agriculture and Horticulture Development Board
EU: European Union
GHG: Greenhouse Gas
GWP: Global Warming Potential
IARC: International Agency for Research on Cancer
LRNI: Lower Reference Nutrient Intake
NDNS: National Diet and Nutrition Survey
NHS: National Health Service
NS-SEC: National Statistics Socio-economic Classification
OECD: Organisation for Economic Co-operation and Development
TPI: Targeted Policy Intervention
WCRF: World Cancer Research Fund
WHO: World Health Organisation
